# Behavioral screening of sleep‐promoting effects of human intestinal and food‐associated bacteria on *Drosophila melanogaster*

**DOI:** 10.1111/gtc.13025

**Published:** 2023-03-28

**Authors:** Taro Ko, Hiroki Murakami, Shunjiro Kobayashi, Azusa Kamikouchi, Hiroshi Ishimoto

**Affiliations:** ^1^ Graduate School of Science Nagoya University Nagoya Aichi Japan; ^2^ Milk Science Research Institute Megmilk Snow Brand Co., Ltd Kawagoe Saitama Japan

**Keywords:** *Bifidobacterium adolescentis*, *Drosophila*, *InR*, insulin, *Lactobacillus plantarum*, sleep

## Abstract

Commensal microbes influence various aspects of vertebrate and invertebrate brain function. We previously reported that *Lactiplantibacillus plantarum* SBT2227 promotes sleep in the fruit fly, *Drosophila melanogaster*. However, how widely the sleep‐promoting effects are conserved in gut bacterial species remains unknown. In this study, we orally administered human intestinal and food‐associated bacterial species (39 in total) to flies and investigated their effects on sleep. Six species of bacteria were found to have significant sleep‐promoting effects. Of these, we further investigated *Bifidobacterium adolescentis*, which had the greatest sleep‐promoting effect, and found that the strength of the sleep effect varied among strains of the same bacterial species. The *B. adolescentis* strains BA2786 and BA003 showed strong and weak effects on sleep, respectively. Transcriptome characteristics compared between the heads of flies treated with BA2786 or BA003 revealed that the gene expression of the insulin‐like receptor (*InR*) was increased in BA2786‐fed flies. Furthermore, a heterozygous mutation in *InR* suppressed the sleep‐promoting effect of BA2786. These results suggest that orally administered sleep‐promoting bacteria (at least BA2786), may act on insulin signaling to modulate brain function for sleep.

## Graphical abstract

Human‐derived and food‐associated bacterial species were orally administered to flies to investigate their effects on sleep. We found that the *Bifidobacterium adolescentis* strain SBT2786 had the greatest sleep‐promoting effect and found that signaling may be required for this effect.

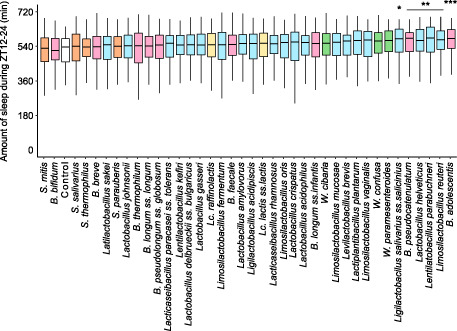

## 1 INTRODUCTION

Sleep or sleep‐like states have been observed in a wide range of animal species (Keene & Duboue, 2018). Sleep is not only necessary for recovery from physical and mental fatigue but also for the proper execution of various brain functions (Rasch & Born, 2013; Raven et al., 2018). Despite sleep having such important functions, people are still not getting sufficient sleep. The prevalence of insomnia in adults is high (at least 10%), although this value varies with study design and the definition of insomnia (Itani et al., 2016; Tiseo et al., 2020). Various sleep‐promoting drugs have been developed to overcome this global sleep‐deprivation problem. However, these solutions themselves cause various problems, such as addiction and dependence, and have considerable side effects (Atkin et al., 2018; Brandt & Leong, 2017). As an alternative to sleep‐promoting drugs, food sources that are less stressful to the body, even when consumed daily, are required.

In recent years, microbes have attracted considerable attention as health enhancers. These include microbes exhibiting anti‐obesity effects (Barathikannan et al., 2019), those associated with reduced risk of cardiovascular disease (Witkowski et al., 2020), immune activation (Shi et al., 2017), and psychological health (Zagórska et al., 2020). However, in mammalian models, it takes an enormous amount of time and research to study the effects of complex gut microbiota on brain function and to select specific bacterial species with sleep‐promoting properties (Panchal & Tiwari, 2017). The fruit fly, *Drosophila melanogaster*, a model organism that is similar to mammals in terms of interactions between the gut microbiota and the host, including immune function and metabolism (Buchon et al., 2014; Wong et al., 2016), serves as an alternative for studying the gut microbiota–brain function axis. Gut microbes influence the brain functions in *D. melanogaster*, including memory (Silva et al., 2020) and foraging decisions (Wong et al., 2017). Moreover, many of the basic features involved in the regulation of sleep in flies and humans are similar, including physiological, pharmacological, endocrine and neuronal regulation, as well as circadian and homeostatic regulation (Huber et al., 2004; Koh et al., 2006; Kume et al., 2005; Ly et al., 2018; Wu et al., 2009; Yuan et al., 2006). Therefore, *D. melanogaster* is a good model organism with simple gut microbiota and neural mechanisms that allow sleep studies to be conducted at the molecular level (Broderick & Lemaitre, 2012; Wong et al., 2011).

We recently identified *Lactiplantibacillus plantarum* SBT2227 (hereafter, referred to as LP2227), which promotes sleep in *D. melanogaster* (Ko et al., 2022). *L. plantarum* is a gram‐positive lactic acid bacterium present in several niches, such as the gastrointestinal tract, feces, meat, vegetables, and dairy substrates (Siezen et al., 2010). However, how a wide range of human intestinal and food‐associated bacterial species affect sleep in flies remains unclear.

In this study, we investigated the effects of 39 species of human intestinal and food‐associated bacteria on sleep in fruit flies. This allowed us to examine the relationship between the effects of sleep and specific bacterial species and genera. Additionally, a combination of transcriptome and behavioral analyses of mutant flies led to the identification of specific genes required for the sleep‐promoting effects of the selected bacteria. These findings suggest that a group of human intestinal or food‐associated bacteria that are ingestible by humans may act on sleep in *Drosophila* and that their actions may target molecular mechanisms common to those in humans. This study opens new avenues for the selection of useful bacteria for inducing sleep and sheds light on the molecular mechanisms that mediate communication between orally ingested bacteria and the host brain.

## 2 RESULTS

### 2.1 Effects of 39 species of human intestinal and food‐associated bacteria on sleep in flies

**FIGURE 1:**
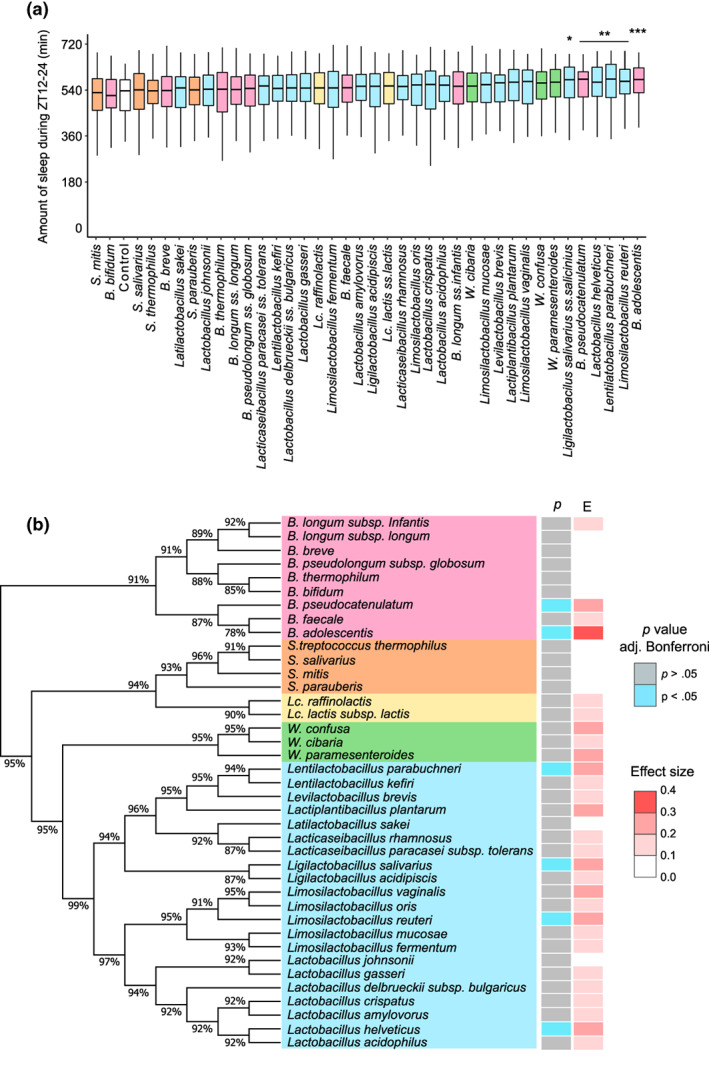
Behavioral screening of the effects of lactic acid bacteria (LAB) and Bifidobacteria on sleep in flies. (a) Amount of sleep between ZT12 and 24 on the third day of oral administration. (b) Relationship between the genetics of each bacterium and the magnitude of the sleep‐promoting effect. Phylogenetic tree of bacteria based on 16S rRNA of their type‐strain is shown, along with the *p* value of the amount of sleep between ZT12 and 24 on the third day and its effect size (e). Bootstrap values are indicated at each node on the phylogenetic tree. The experiments on *n* = 16 flies/group/experiment were repeated six times and *n* = 96 flies/group were obtained. For statistical analyses, the amount of sleep was compared with that in the control group. Statistical difference was determined using the Wilcoxon–Mann–Whitney test and adjusted with the Bonferroni correction. *p < .05; **p < .01; ***p < .001.

To select human intestinal and food‐associated bacteria that exhibit sleep‐promoting effects, 30 lactic acid bacteria (LAB) and nine bifidobacteria were individually administered orally to flies. The LABs evaluated in this study belonged to the following genera: *Lacticaseibacillus*, *Lactiplantibacillus*, *Lactobacillus*, *Latilactobacillus*, *Lentilactobacillus*, *Levilactobacillus*, *Ligilactobacillus*, *Limosilactobacillus*, *Weissella*, *Lactococcus*, and *Streptococcus*. In this study, all bacterial cells used for screening were obtained from the same amount of culture media. Analysis of the amount of sleep at night, which is the primary sleeping time for *Drosophila*, revealed that six bacterial species significantly increased the amount of sleep compared with that in the control group (Figure 1a). Four of the six species were identified as *Limosilactobacillus reuteri*, *Lentilactobacillus parabuchneri*, *Lactobacillus helveticus*, and *Ligilactobacillus salivarius* subsp. *salicinius* was formerly classified as a *Lactobacillus* (Zheng et al., 2020). The remaining two species were *Bifidobacterium adolescentis* and *Bifidobacterium pseudocatenulatum*.

To compare the degree of sleep‐promoting effects between different bacterial species, effect sizes were calculated for the administration of each bacterium and plotted on a tree diagram based on the 16S rRNA of each bacterial strain (Figure 1b). The highest effect size was observed for the *B. adolescentis* strain SBT2786 (hereafter, referred to as BA2786) (*r* = 0.31). *B. pseudocatenulatum*, another member of *Bifidobacterium* that significantly increases sleep, showed a weak effect size; however, the remaining seven bifidobacteria did not show significant promotion of night‐time sleep. The remaining four lactobacilli that significantly increased sleep were not concentrated in specific branches of the former *Lactobacillus* branch. In contrast, none of the four *Streptococcus*, two *Lactococcus*, or three *Weissella* species examined had any significant effect on the sleep of flies. These results suggest that the sleep‐promoting effects of 39 human intestinal or food‐associated bacteria are related to the genus. Based on these screening results, we focused on BA2786 to investigate its sleep and waking effects in more detail.

### 2.2 Characteristics of the effect of *B. adolescentis* BA2786 on sleep and wakefulness

**FIGURE 2:**
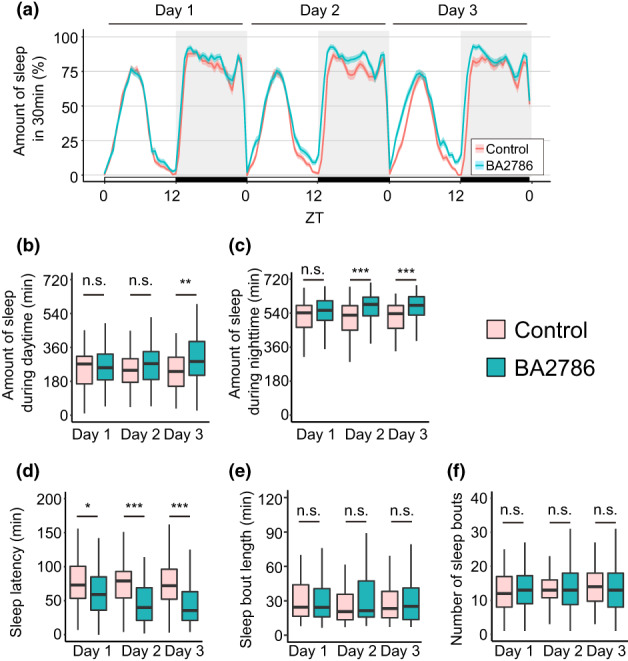
Oral administration of BA2786 increased the amount of sleep at the onset of the night‐time and decreased the sleep latency. (a) Sleep patterns of flies fed control (red) or BA2786 (green) food. Sleep patterns are indicated with mean ± SEM. (b) Amount of sleep during the daytime (ZT0–12), (c) amount of sleep during the night‐time (ZT12–24), (d) sleep latency, (e) sleep bout length, and (f) number of sleep bouts during the night‐time (*n* = 96 for each group). The Wilcoxon–Mann–Whitney test was used for statistical analysis and adjusted with the Bonferroni correction. *p < .05; **p < .01; ***p < .001; n.s., not significant.

The daytime sleep amount of the flies fed BA2786 increased only on the third day, and the sleep amount at night increased on the second and third days (Figure 2a–c). Sleep latency was significantly reduced in BA2786‐fed flies on all 3 days (Figure 2d). The amount of sleep was expressed as the sum of the length of each sleep episode (sleep bouts). There were no significant differences between the BA2786 and control groups in the length and number of sleep bouts in response to a significant increase in total sleep amount during the night (Figure 2e,f). To help understand this contradictory phenomenon, we verified the cumulative sleep amount for each range of sleep bout length, from the shortest to the longest (Figure S1). Cumulative sleep amount from sleep bout length of less than 40 min, including over half of sleep bouts (Figure 2e), did not differ between the two groups. However, the BA2786‐treated group showed lower integrated values up to the sleep bout length of approximately 300 min and higher values after 300 min, compared with that in the control group. This result suggests that the increased amount of total night‐time sleep was due to an increase in the longer fraction of the sleep bout length. Longer sleep bouts indicate higher sleep consolidation, whereas shorter sleep bouts indicate sleep fragmentation (Koh et al., 2006). These results suggest that BA2786 increases the total amount of night‐time sleep by consolidating the sleep.

In addition, BA2786 was killed by heating at 65°C for 1 h (<100 cfu/ml; below the detection limit) and administered to flies. The amount of sleep at night and the sleep latency on the third day, measured as representative values of the sleep‐promoting effects of BA2786, were significantly increased and shortened, respectively (Figure S2). These results indicate that BA2786 need not be alive to promote sleep in flies.

**FIGURE 3:**
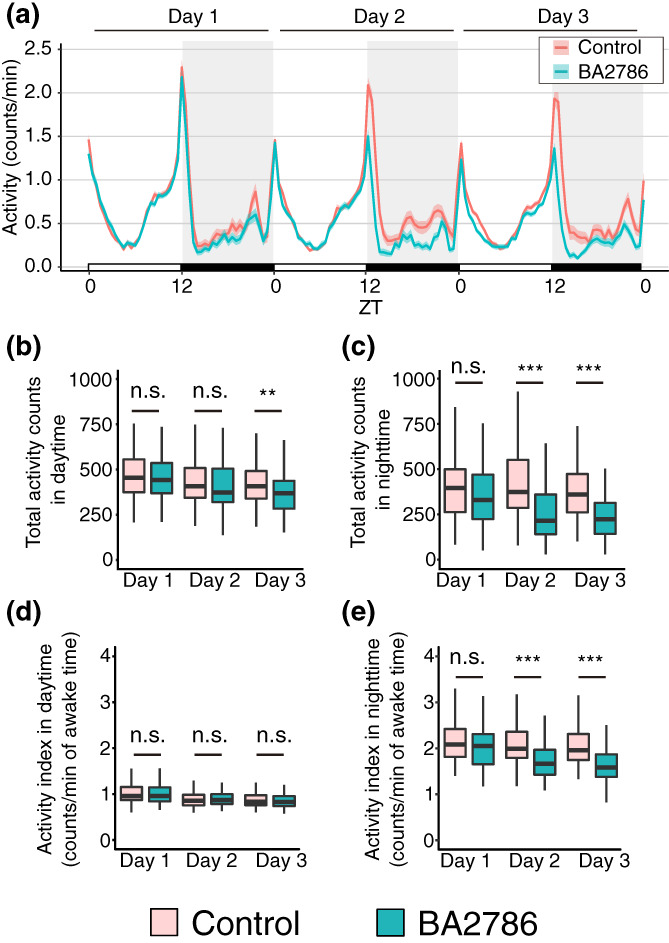
Wakefulness was altered in BA2786‐fed flies. (a) Activity patterns of flies on control (red) or BA2786 (green) food. (b) Activity counts during the daytime (ZT0–12), and (c) during the night‐time (ZT12–24). (d) Activity index during the daytime, and (e) during the night‐time (*n* = 96 for each group). The Wilcoxon–Mann–Whitney test was used for statistical analysis and adjusted with the Bonferroni correction. *p < .05; **p < .01; ***p < .001; n.s., not significant.

Next, we examined the effect of BA2786 on wakefulness. This is because the sleep‐promoting effects of BA2786 may be caused by factors, such as reduced motor function. Activity pattern plots suggested that administration of BA2786 decreased the fly activity (Figure 3a). The active counts were significantly decreased in the daytime on the third day and in the night‐time on the second and third days (Figure 3b,c). Next, we examined another parameter of the awake behavior: the waking activity index (activity counts per waking minute). The feeding of BA2786 caused no significant change in the waking activity index during the daytime but decreased the index during the night‐time on the second and third days (Figure 3d,e). These results suggest that the decrease in total activity counts at night induced by BA2786 is not only due to a decrease in awakening time but also due to a decrease in the activity level after awakening. Furthermore, no change in the waking activity index during the daytime was observed, which is the main active period, suggesting that BA2786 does not disturb the motor function of the flies.

### 2.3 Characteristics of the effect of *B. adolescentis* BA003 on sleep and wakefulness

**FIGURE 4:**
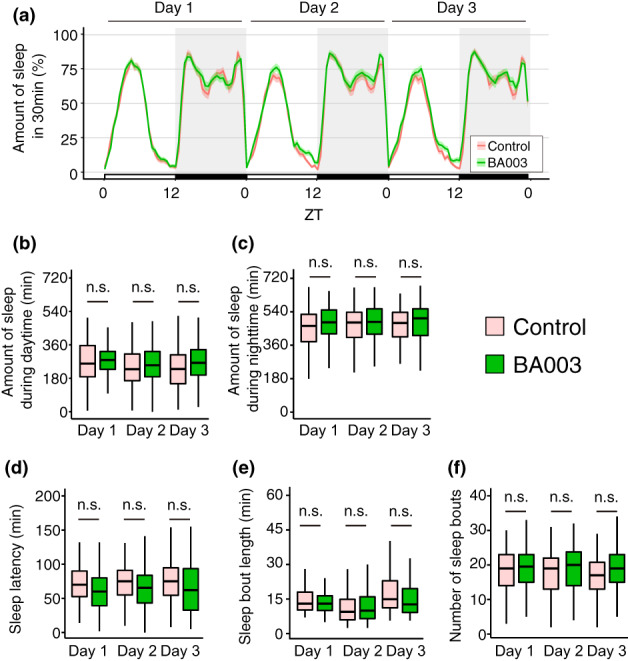
Oral administration of BA003 did not affect the fly sleep. (a) Sleep patterns of flies fed control (red) or BA003 (green) food. Sleep patterns are indicated with mean ± SEM. (b) Amount of sleep during the daytime (ZT0–12), (c) amount of sleep during the night‐time (ZT12–24), (d) sleep latency, (e) sleep bout length, and (f) number of sleep bouts during the night‐time (*n* = 94–95 for each group). The Wilcoxon–Mann–Whitney test was used for statistical analysis and adjusted with the Bonferroni correction. n.s., not significant.

**FIGURE 5:**
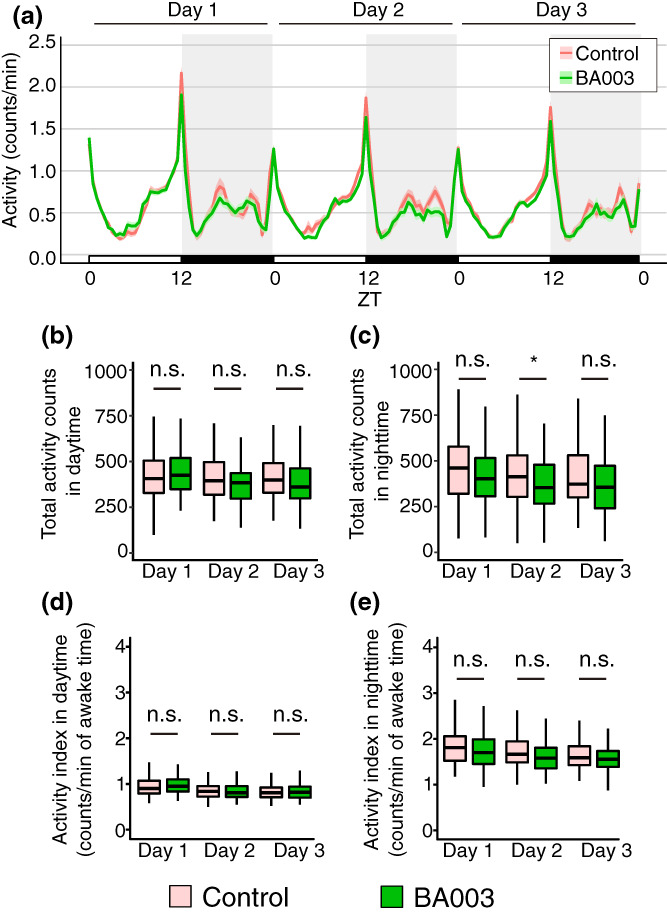
Wakefulness was mostly unaltered in flies fed BA003. (a) Activity patterns of flies fed control (red) or BA003 (green) food. (b) Activity counts during the daytime (ZT0–12), and (c) during the night‐time (ZT12–24). (d) Activity index during the daytime, and (e) during the night‐time (*n* = 94–95 for each group). The Wilcoxon–Mann–Whitney test was used for statistical analysis and adjusted with the Bonferroni correction. *p < .05; n.s., not significant.

Different strains of the same bacterial species may have different quantitative and qualitative effects on the host animal (Hill et al., 2014; McFarland et al., 2018). Therefore, we investigated the effects of BA003, a variant of *B. adolescentis* BA2786, on sleep. BA003, similar to BA2786, is a strain of *B. adolescentis* isolated from human feces. In contrast to BA2786, which significantly increased the amount of night‐time sleep in flies, no significant changes were detected in the sleep of flies after the administration of BA003 (Figure 4a). No statistical differences were detected in the amount of daytime and night‐time sleep, sleep latency, sleep bout length, or the number of sleep bouts in flies administered BA003 (Figure 4b–f). Similarly, BA003‐fed flies showed no apparent effect on the activity patterns (Figure 5a). Although a slight decrease in night‐time activity on the second day was detected in BA003‐fed flies, other parameters such as active counts and activity index, both during the daytime and night‐time did not change significantly (Figure 5b–e). Taken together, the administration of BA003 had little or no effect on sleep. This was possibly due to the flies simply not ingesting BA003. However, there was no significant difference in food consumption between the control, BA2786, and BA003 (Figure S3). This finding indicates that although BA003 belongs to the same species, it has a lesser effect on fly sleep when compared with the sleep‐promoting effects of BA2786.

### 2.4 Changes in gene expression due to bacterial feeding

**FIGURE 6:**
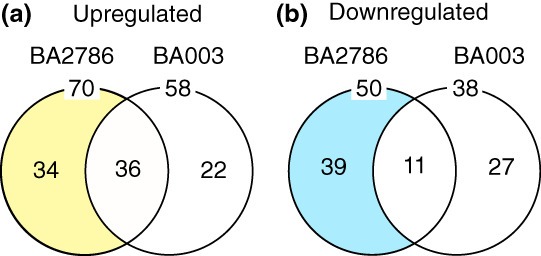
Transcriptome‐based analysis of the effects of BA2786 or BA003 in the fly head. (a,b) Venn diagram showing the number of genes whose expression level was changed relative to that in the control group. Genes with increased expression levels are shown in (a) and genes with decreased expression levels are shown in (b).

The phenotypic differences in genetically related bacterial strains could be due to the differences in gene expression levels in flies after feeding the different strains. Based on this assumption, we explored the changes in gene expression associated with the sleep‐promoting effects of BA2786 in flies. We compared the transcriptome characteristics of heads from BA2786‐ or BA003‐fed flies with those of unfed flies using RNAseq. When compared with the unfed flies, the number of genes whose expression was increased more than 2‐fold (*p* < .05, chi‐square test) was 70 in the BA2786‐fed flies and 58 in the BA003‐fed flies. In contrast, genes with reduced expression levels were 50 in the BA2786‐fed flies and 38 in the BA003‐fed flies (Figure 6). Additionally, the number of genes whose expression was upregulated and downregulated only in BA2786‐fed flies was 34 and 39, respectively (Figure 6, Tables 1 and 2). One of the upregulated genes in BA2786‐fed flies was insulin‐like receptor (*InR*), which was also upregulated in flies fed LP2227, a *L*. *plantarum* strain that promotes sleep in fruit flies (Ko et al., 2022) (Table S1). Therefore, we focused on *InR* and examined the effects of BA2786 on sleep in an *InR* hypomorphic mutant.

**TABLE 1:** Genes upregulated by the administration of BA2786 but not by that of BA003.

Gene		mRNA expression level (relative to that in the control group)	
Name	FLYBASE no.	BA2786	BA003
Cyclin B	FBgn0000405	7.2	2.7
G protein beta‐subunit 13F	FBgn0001105	2.4	1.1
Phosphoenolpyruvate carboxykinase 1	FBgn0003067	2.2	1.7
Proliferating cell nuclear antigen	FBgn0005655	8.0	3.0
Odorant‐binding protein 28a	FBgn0011283	2.5	1.2
Cyclin B3	FBgn0015625	3.0	2.2
Pinocchio	FBgn0016926	2.5	−1.0
Odorant‐binding protein 56a	FBgn0034468	2.2	1.7
Zye	FBgn0036985	2.2	1.2
Meiosis regulator and mRNA stability factor 1	FBgn0039972	2.6	2.2
Attacin‐B	FBgn0041581	3.9	1.8
Claspin	FBgn0052251	3.4	1.3
Alpha‐Tubulin at 67C	FBgn0087040	2.9	−1.1
Uncoordinated 115b	FBgn0260463	2.0	1.0
Pre‐mod(mdg4)‐C	FBgn0266170	5.3	1.6
Penduline	FBgn0267727	6.7	2.6
Insulin‐like receptor	FBgn0283499	2.1	1.0
Uncharacterized protein	FBgn0028855	2.1	1.9
Uncharacterized protein	FBgn0033374	2.3	1.6
Uncharacterized protein	FBgn0034440	2.3	−1.9
Uncharacterized protein	FBgn0036979	5.3	3.3
Uncharacterized protein	FBgn0037844	3.6	1.8
Uncharacterized protein	FBgn0038099	3.4	2.0
Uncharacterized protein	FBgn0051773	2.5	1.6
Uncharacterized protein	FBgn0085194	2.3	1.9
Uncharacterized protein	FBgn0264742	2.9	1.0
Uncharacterized protein	FBgn0266434	3.2	1.7
Uncharacterized protein	FBgn0266540	2.1	−2.6
Uncharacterized protein	FBgn0267964	3.1	1.2
Small nucleolar RNA Me18S‐G1358	FBgn0286758	57.1	−1.1
Long non‐coding RNA on the X 1	FBgn0019661	2.7	1.3
Long non‐coding RNA:CR44347	FBgn0265447	3.2	1.1
Long non‐coding RNA:CR45447	FBgn0267003	4.7	3.9
28S ribosomal RNA	FBgn0267504	9.5	−1.7

**TABLE 2:** Genes downregulated by the administration of BA2786 but not by that of BA003.

Gene		mRNA expression level (relative to that in the control group)	
Name	FLYBASE no.	BA2786	BA003
Small nuclear RNA U2 at 34AB a	FBgn0004191	−6.6	−1.3
Corto	FBgn0010313	−2.1	1.1
Chico	FBgn0024248	−2.0	1.0
Turandot A	FBgn0028396	−8.0	−1.0
Chemosensory protein A 7a	FBgn0029948	−4.3	−1.7
Turandot M	FBgn0031701	−8.2	1.7
Hyccin	FBgn0034269	−2.6	1.0
Immune induced molecule 23	FBgn0034328	−2.4	−2.0
Diedel	FBgn0039666	−3.3	2.0
Prolyl‐4‐hydroxylase‐alpha NE1	FBgn0039780	−4.4	−3.2
Mediator complex subunit 26	FBgn0039923	−2.5	1.1
Turandot X	FBgn0044810	−2.2	1.3
Turandot C	FBgn0044812	−17.5	1.0
Eukaryotic translation initiation factor 4E homologous protein	FBgn0053100	−2.1	1.0
p24‐related‐2	FBgn0053105	−19.1	−1.1
Nimrod C4	FBgn0260011	−2.6	−1.3
Glycerol‐3‐phosphate dehydrogenase 3	FBgn0263048	−2.4	−1.7
Pre‐mod(mdg4)‐K	FBgn0266173	−3.2	−2.6
Nicotinic acetylcholine receptor alpha4	FBgn0266347	−2.4	1.1
Uncharacterized protein	FBgn0000092	−2.3	−1.7
Uncharacterized protein	FBgn0034515	−4.1	−1.6
Uncharacterized protein	FBgn0035228	−4.5	−1.1
Uncharacterized protein	FBgn0039945	−2.2	1.4
Uncharacterized protein	FBgn0040637	−2.1	−1.4
Uncharacterized protein	FBgn0050154	−2.8	−2.0
Uncharacterized protein	FBgn0051806	−12.6	−2.1
Uncharacterized protein	FBgn0051875	−6.2	−1.1
Uncharacterized protein	FBgn0085193	−7.4	−1.4
Uncharacterized protein	FBgn0085246	−8.6	−2.9
Uncharacterized protein	FBgn0259202	−4.0	−1.1
Uncharacterized protein	FBgn0259699	−2.9	−1.7
Uncharacterized protein	FBgn0259709	−11.9	−1.4
Uncharacterized protein	FBgn0259933	−2.8	−1.8
Uncharacterized protein	FBgn0259989	−6.5	−2.7
Uncharacterized protein	FBgn0260222	−5.0	−1.7
Uncharacterized protein	FBgn0264743	−3.1	1.0
Long non‐coding RNA:CR33987	FBgn0053987	−4.1	−1.3
Long non‐coding RNA:CR45204	FBgn0266731	−5.2	−1.6
Pre‐ribosomal RNA	FBgn0267507	−318.9	−1.7

### 2.5 Requirement of the insulin‐like receptor for the sleep‐promoting effect of BA2786

**FIGURE 7:**
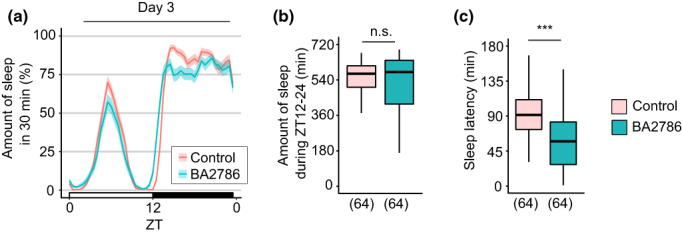
The effect of *InR* in the sleep‐promoting effects of BA2786. (a) Sleep patterns on the third day of flies fed control (red) or BA003 (green) food. Sleep patterns are indicated as mean ± SEM. (b) Amount of sleep during ZT12–24 and (c) sleep latency on the third day. The sample sizes are shown below each graph. The Wilcoxon–Mann–Whitney test was used for statistical analysis. ***p < .001; n.s., not significant.

In previous studies, flies that were *InR* heterozygous mutants and those that ubiquitously expressed a dominant‐negative form of *InR* showed a typical sleep pattern of daytime and night‐time sleep, which was similar to the basal sleep phenotypes of control flies (Cong et al., 2015; Yamaguchi et al., 2022). To investigate the involvement of *InR* in the sleep‐promoting effects of BA2786, we administered BA2786 to a heterozygous *InR* mutant (*InR[E19]/+*). Surprisingly, *InR[E19]/+* flies showed no increase in the night‐time sleep following oral administration of BA2786 (Figure 7a,b). Intriguingly, oral administration of BA2786 significantly shortened the sleep latency even in *InR[E19]*/+ flies (Figure 7c). These results suggest that although the heterozygous *InR* mutation has no drastic effect on the amount of night‐time sleep, it affects at least a part of the sleep‐promoting effects of BA2786.

## 3 DISCUSSION

In this study, we evaluated the sleep‐promoting effect of 39 species of LABs and bifidobacteria using a previously established high‐throughput system for sleep assessment (Hendricks et al., 2000; Shaw et al., 2000). *Streptococcus*, *Lactococcus*, and *Weissella* did not show any significant sleep‐promoting effects. Two *Weissella* species slightly, but not significantly, increased the amount of sleep, and thus might have a sleep‐promoting effect that was not observed in our large‐scale screening. On the contrary, all species of *Streptococcus* and *Lactococcus* did not alter the amount of fly sleep, suggesting that the sleep‐promoting effect may be weak.

In contrast, *B. adolescentis* (BA2786), *B. pseudocatenulatum*, *L. parabuhneri*, *L. salivarius*, *L. reuteri*, and *L. helveticus* showed significantly increased sleep‐promoting effect. Interestingly, these bacteria are not genetically close, suggesting that the genetic relationship between the bacterial species is unlikely to be correlated with the magnitude of the sleep‐promoting effect. Consistently, among *Bifidobacterium* species, BA2786 (*B. adolescentis*) promoted sleep, whereas *B. bifidum* did not. Even within the same species, BA2786 promoted sleep, whereas BA003 did not. Taken together, the sleep‐promoting effect of bacteria is widely distributed among different bacterial genera, species, and strains, rather than being an evolutionarily conserved phenotype in a specific bacterial group.

*Bifidobacterium adolescentis* strain BA2786 promoted night‐time sleep. BA2786 had a lesser effect on the daytime behavior in both sleep and wakefulness when compared with that on the night‐time behavior. Furthermore, BA2786 significantly shortened the sleep latency but did not alter the length and number of sleep bouts during the night or the waking activity index during the daytime. These characteristics are similar to the sleep responses of flies after the administration of LP2227 (Ko et al., 2022). Fly sleep can be categorized into several phenomena, such as morning or evening sleep, the onset of night‐time sleep, the second half of night‐time sleep, and falling asleep, each of which are regulated by distinct mechanisms (Agosto et al., 2008; Gmeiner et al., 2013; Liang et al., 2019). The induction of similar sleep phenotypes upon administration of BA2786 and LP2227 suggests that these two bacteria may act on a common part of the *Drosophila* sleep regulation mechanism, or may be coincidental due to the regulation of different mechanisms. Furthermore, looking at the similarity of phenotypes by BA2786 and LP2227 from the bacterial side suggests the following possibilities: one, the active substance is different for each bacterium, and the other, the active substance is the same even if the bacterial species are different. The identification of sleep‐promoting substances would be of great interest to future studies; however, this study focused only on the mechanism of sleep regulation in flies by BA2786.

In addition, heat‐killed BA2786 promoted sleep in flies, which indicates that BA2786 is probably not a probiotic. The prebiotic effect of BA2786—its effect via the existing gut bacteria—was not tested in this study. Flies with artificially removed gut bacteria sleep slightly more (Selkrig et al., 2018; Silva et al., 2020), which is indicative of the inhibition of sleep by the fly gut microbiota. BA2786 may, therefore, have the potential to promote sleep, at least the night‐time sleep, by overcoming the sleep‐inhibiting effects of existing gut bacteria in flies, either by acting directly on the gut bacteria or by acting on biological pathways in flies in which the gut bacteria have a role. The fact that the sleep‐promoting effect of BA2786 appeared from the second day after administration may also be indicative of this prebiotic effect. To understand the mechanism of action of BA2786 and reason for the delay in the onset of the sleep phenotype, it is necessary to understand, at a molecular level, the compounds responsible for the action and the targeted tissues or cells.

Cong et al. reported that the *InR* mutant *Drosophila* exhibited reduced sleep than wild‐type flies and expression of a gain‐of‐function form of *InR* derived from the *GMR28E02‐gal4* line, a Gal4 driver line for *InR*, caused more sleep than the parental strains (Cong et al., 2015). Yamaguchi et al. reported that the expression of a dominant‐negative form of the *InR* in anterior dorsal neuron group 1, one of the circadian neurons in the brain, and pars intercerebralis, one of the output regions of the circadian neurons, causes less sleep (Yamaguchi et al., 2022). These results supported the notion that the increase in *InR* expression observed with BA2786 may contribute to the promotion of sleep in flies. The insulin pathway is very similar in *Drosophila* and mammals (Álvarez‐Rendón et al., 2018). For example, in both human and fly insulin receptors, an exchange of amino acids at the corresponding positions causes growth retardation (Brogiolo et al., 2001). Based on this, we hypothesized that BA2786 acts on insulin signaling in humans. Interestingly, among the sleep‐promoting effects of BA2786, the significant increase in sleep amount, which was observed in wild‐type flies, disappeared in the *InR* heterozygous mutant, but the shortening of sleep latency did not. This indicates that *InR* is involved, either directly or indirectly, in increasing the amount of sleep but not in shortening the sleep latency. This was further validated as the differences between wild‐type and *InR* heterozygous mutant flies showed no significant effect on the decrease in the sleep latency due to BA2786 administration (data from the third night in Figures 2d and 7b were compared using two‐factor aligned rank transform ANOVA, *F* (1, 316) = 0.067, *p* = .80). This suggested that the decrease in sleep latency due to BA2786 administration was not significantly different between wild‐type and *InR* heterozygous mutant flies. Thus, it is likely that the shortening of sleep latency is regulated by a mechanism independent of *InR*.

The effects of *InR* on sleep vary depending on the site of expression (Yamaguchi et al., 2022). Furthermore, insulin signaling differentially influences the sleep/awake behavior through multiple pathways, including the promotion of daytime activity by FOXO, and the reduction of sleep duration and sleep consolidation at night by S6K (Metaxakis et al., 2014). Therefore, to elucidate the mechanism of the sleep‐promoting effects of BA2786, detailed studies on the effects and downstream signals of *InR* on sleep in each brain region are needed. Additionally, transcriptome analysis revealed that *InR* expression was upregulated and its target molecule, *chico*, was downregulated in BA2786‐fed flies. In contrast, in LP2227‐fed flies, the expression of *InR* was upregulated, but that of *chico* did not change. Despite these differences, the effects of BA2786 and LP2227 on the fly sleep behavior were similar. Chico is phosphorylated by *InR* and transmits downstream signals (Poltilove et al., 2000). Therefore, even if the expression of *chico* was reduced by BA2786 administration, it is possible that a sufficient amount was available for downstream signaling. Indeed, there are other possibilities, such as the regulation of the sleep behavior by *InR* through pathways independent of *chico*. It is necessary to clarify how *chico* is involved in sleep regulation by examining the effects of downstream signals of *InR*.

Comparison of the transcriptomes of flies fed BA2786 and BA003 showed that, in addition to *InR*, the expression of genes related to the cell cycle, including *cyclin B*, *cyclin B3*, *proliferating cell nuclear antigen*, and *claspin*, was increased in BA2786‐fed flies. Therefore, it is possible that these cell cycle‐related genes regulate sleep and that they are involved in the sleep‐promoting effects of BA2786. Although another cell cycle regulator, cyclin A, has been reported to promote sleep (Rogulja & Young, 2012), no change in its expression was detected in this study.

In contrast, genes belonging to the *Turandot* family were downregulated in the BA2786‐fed flies. The *Turandot* family genes are considered humoral stress‐response factors. For example, *Turandot A* is upregulated by a wide range of stresses, including heat stress, bacterial infection, and restraint stress (Ekengren & Hultmark, 2001; Seong et al., 2020). In humans, sleep loss elevates the stress hormone cortisol (Leproult & Van Cauter, 2010). In contrast, getting enough sleep lowers stress hormone levels. Therefore, the decrease in the levels of *Turandot* family genes by BA2786 administration, observed in this study, may be due to the BA2786‐mediated sleep promotion.

In this study, we systematically investigated genes that may be involved in the sleep‐promoting effects of strains of sleep‐promoting and non‐sleep‐promoting bacteria. Although the effects of bacteria on sleep behavior of flies appear similar, there are cases in which the mechanisms of action may differ. Future research focusing on the insulin signaling found in this study will allow a deeper understanding of the bacterial mechanism of action on sleep. There are potential limitations to this study. First, the bacterial doses were aligned with the amount of culture medium; it is, therefore, not possible to determine whether the sleep‐promoting effect was due to the quality or number of bacteria. Second, only female flies were tested; because males sleep better than females, it was difficult to assess the sleep‐promoting effects of the bacteria. It is known that the insulin signaling activity differs with sex (Millington et al., 2021). Future studies focused on insulin signaling may address this limitation.

## 4 EXPERIMENTAL PROCEDURES

### 4.1 Fly strains and rearing conditions

*Canton‐S* ^*2202u*^ *w*as obtained from Dr. Sakai (Tokyo Metropolitan University, Japan) and *InR* ^*E19*^ */TM2* (RRID:BDSC_9646) was obtained from the Bloomington *Drosophila* Stock Center (Indiana University Bloomington, Bloomington, IN). *InR* ^*E19*^ */TM2* was crossed with *Canton‐S* ^*2202u*^ to obtain the heterozygous mutant, *InR* ^*E19*^ */+*. All flies were reared and maintained at 24 ± 1°C with a relative humidity of 60 ± 3% on standard cornmeal yeast food (50 g/L glucose, 45 g/L yeast, 40 g/L corn flour, 8 g/L agar, 4 mL/L propionic acid, and 3 mL/L methyl 4‐hydroxybenzoate) in a 12 h light/12 h dark (12 h L/D) cycle. Virgin female flies were used in all the experiments. Flies were collected under CO_2_ anesthesia and maintained in food vials (15–20 flies per vial) until further experiments.

### 4.2 Preparation of bacterial samples

All bacterial strains were obtained from Megmilk Snow Brand Co., Ltd. (Tokyo, Japan) and were cultured under the conditions described in Table S2. Cultured bacteria were collected by centrifugation (7000 × *g*, 20 min, 4°C) and washed two times with sterilized saline (0.9% NaCl). For behavioral screening, bacteria were cultured in 240 ml of medium. To prepare frozen stocks of the bacteria, bacterial pellet was resuspended in trehalose solution (12.5% final) and concentrated to a final volume of one‐tenth of the culture medium (24 ml). The bacterial suspension, dispensed into six 4 ml cryotubes, was incubated at 4°C for 2–5 h and then rapidly frozen in liquid nitrogen for storage at −80°C until further use. For other experiments, the bacterial pellets washed with sterilized saline were washed again with distilled water, lyophilized, and stored at −80°C until experiments.

### 4.3 Sleep analysis

Fly sleep was measured using the *Drosophila* activity monitoring system (Trikinetics, Waltham, MA) as previously described (Ko et al., 2022). Four‐day‐old virgin female flies were transferred to a glass tube (5 × 65 mm^2^) with control or test food on one end of the tube, and the other end was sealed with a cotton plug after loading the fly. The flies were allowed to acclimatize to the new environment overnight, and locomotor activity was measured for 3–4 days at 25 ± 1°C under a 12 h light/dark cycle.

For the behavioral screening and administration of heat‐killed BA2786, the control food contained trehalose (6.25%), sucrose (5.0%), and Bacto‐agar (1.0%). The test food contained a mixture of bacterial suspensions from the control food. Briefly, equal amounts of the bacterial suspension containing trehalose (12.5%) and sucrose agar solution (10.0% sucrose, 2.0% Bacto‐agar) were mixed. Heat‐killed BA2786 was obtained by incubating a bacterial suspension containing trehalose at 65°C for 1 h (HB‐80, TAITEC Co., Saitama, Japan).

For other experiments, the control food consisted of sucrose (5.0%) and Bacto‐agar (1.0%), and the test food consisted of the control food along with the lyophilized bacteria. The concentration of lyophilized bacteria was approximately 1%.

Locomotor data were collected in 1 min bins, and inactivity for at least 5 min was defined as a sleep bout. All behavioral parameters were calculated and statistically analyzed for individual flies using the R package Rethomics (Geissmann et al., 2019). Dead animals were excluded from the analysis. The amount of sleep was calculated by adding the duration of all sleep events within the time of interest. Sleep latency was defined as the time from ZT12 (start of the night‐time) to the detection of the first sleep event. The duration of a sleep event detected during the time of interest was used as the sleep bout length. The number of sleep events detected during the time of interest was defined as the number of sleep episodes. The number of times a fly crossed the infrared beam during the time of interest was used as an activity count. The daytime or night‐time activity index was calculated by dividing the number of activity counts by the total waking time (min) for each time period. Locomotor data were analyzed from the third day, unless otherwise stated.

### 4.4 Measuring food consumption

Fly food consumption of BA2786 or BA003 and control food was measured as previously reported, with some modifications (Ko et al., 2022; Shell et al., 2018; Wu et al., 2020). Briefly, blue dye (Blue No. 1, Fujifilm Wako Pure Chemicals Corporation, Osaka, Japan) was added to the foods at 1% w/v, and the foods were filled in feeder cups (10 μl tip, Molecular BioProducts, San Diego, CA). The feeder cups were set in holes drilled in the lids of conical tubes (50 ml conical tube, Greiner, Kremsmünster, Austria), and 10 flies were placed in each tube. After 6 h of feeding on the colored food, the flies were collected and smashed in 1 mL of water to extract the blue dye from their bodies. Thereafter, 1 ml of water was added to the conical tube and the dye was extracted from feces on the surface of the tube. These dye extracts were mixed in equal amounts and the absorbance was measured at 630 nm using a spectrophotometer (Infinite M Plex, Tecan, Switzerland).

### 4.5 Phylogenetic analysis

The 16S rRNA sequences used for sequence alignment and phylogenetic analysis were obtained from the NCBI database (https://www.ncbi.nlm.nih.gov/nuccore/). The 16S rRNA sequences of the type strains of the bacterial species evaluated during sleep are tabulated in Table S2. Phylogenetic trees were computed with MEGA X (Kumar et al., 2018) using the neighbor‐joining method.

### 4.6 RNA sequencing analysis

Heads were collected between ZT12 and 13, 4 days after the start of the sleep experiment, from flies fed control or test food (BA2786 or BA003). Total RNA was extracted from the heads using the RNeasy mini kit (Qiagen, Hilden, Germany), according to the manufacturer's protocol. RNA sequencing libraries were constructed and sequenced by Macrogen Inc. (Seoul, South Korea), using the Illumina NovaSeq6000 system (San Diego, CA). Read fragments were mapped to the *D. melanogaster* genome NCBI GCF_000001215.4_Release_6_plus_ISO1_MT and assembled using StringTie (Pertea et al., 2015). edgeR (Robinson et al., 2010) was used to compare the RNA expression, and the exact test for negative binomial distribution was applied. For the genes expressed in all groups, expression levels were compared in each of the two groups, and genes with more than 2‐fold change in expression and *p* < .05 were analyzed.

### 4.7 Quantification and statistical analysis

All statistical analyses were performed using the R Studio version 1.4.1106 (www.r-project.org). All data were checked for normality using the Shapiro–Wilk test, and appropriate statistical methods were applied. When Welch's *t*‐test or Wilcoxon–Mann–Whitney test was repeated, the *p*‐values were adjusted using the Bonferroni method. Statistical significance was set at *p* < .05. The boxes in the box and whisker plots represent the median and interquartile range (the distance between the first and third quartiles), and the whiskers represent the highest and lowest data points, excluding outliers. To evaluate the sleep‐promoting effect, the effect size, *r*, was calculated from the *z*‐value of the Wilcoxon–Mann–Whitney test using the following formula: *r* = z/√n.

## AUTHOR CONTRIBUTIONS

Taro Ko, Hiroki Murakami, and Hiroshi Ishimoto designed the research; Taro Ko, Hiroki Murakami, Shunjiro Kobayashi, and Hiroshi Ishimoto performed the research; Taro Ko, Hiroki Murakami, Shunjiro Kobayashi, and Hiroshi Ishimoto analyzed the data; Taro Ko, Azusa Kamikouchi, and Hiroshi Ishimoto wrote the manuscript; and Azusa Kamikouchi and Hiroshi Ishimoto reviewed and edited the manuscript. All authors have read and approved the manuscript.

## CONFLICTS OF INTEREST STATEMENT

Taro Ko, Hiroki Murakami, and Shunjiro Kobayashi were employees of the Megmilk Snow Brand Co., Ltd. The other authors declare no conflicts of interest.

## Supporting information


**Figure S1.** Accumulated sleep amount for each range of sleep bout length during ZT12–24 on the third day. *n* = 96 for each group.

**Figure S2.** Heat‐killed SBT2786 promoted sleep in flies. The effects of heat‐killed SBT2786 were tested in flies. (A) Sleep patterns of flies on the third day. Sleep patterns are indicated as mean ± SEM. (B) Amount of sleep during ZT12–24 on day 3 and (C) sleep latency on the third day. The sample sizes are shown below each graph. The Wilcoxon–Mann–Whitney test was used for statistical analysis. *p < .05; ***p < .001.

**Figure S3.** Food consumption by flies of BA2786 and BA003 food was not different from that of the control food. The amount of food consumed by flies in 6 h was measured for each type of food. Bars represent mean ± SD. *n* = 5 for each group. The Tukey–Kramer test was applied for statistical analysis. n.s.; not significant.

**Table S1.** Genes whose expression levels were upregulated or downregulated in flies treated with LP2227.
**Table S2.** List of bacterial strains used in the study.
