# Supplementary material for: Behavioral screening of sleep‐promoting effects of human intestinal and food‐associated bacteria on Drosophila melanogaster
Source: Genes Cells. 2023 Mar 28;28(6):433–46. doi: 10.1111/gtc.13025 (PMC11447928; doi:10.1111/gtc.13025)
Supplement: Supplementary file 1 — Figure S1. Accumulated sleep amount for each range of sleep bout length during ZT12–24 on the third day. n = 96 for each group. [file GTC-28-433-s004.pdf]

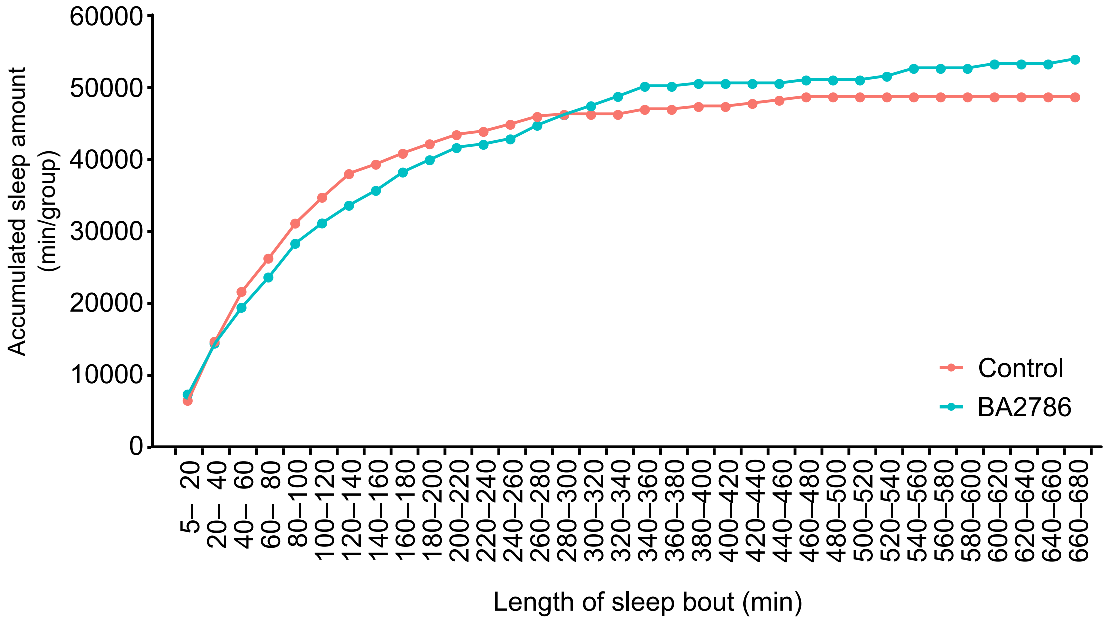

Figure S1. Accumulated sleep amount for each range of sleep bout length during ZT12–24 on the third day. $n = 96$ for each group.
