# Supplementary material for: Behavioral screening of sleep‐promoting effects of human intestinal and food‐associated bacteria on Drosophila melanogaster
Source: Genes Cells. 2023 Mar 28;28(6):433–46. doi: 10.1111/gtc.13025 (PMC11447928; doi:10.1111/gtc.13025)
Supplement: Supplementary file 2 — Figure S2. Heat‐killed SBT2786 promoted sleep in flies. The effects of heat‐killed SBT2786 were tested in flies. (A) Sleep patterns of flies on the third day. Sleep patterns are indicated as mean ± SEM. (B) Amount of sleep during ZT12–24 on day 3 and (C) sleep latency on the third day. The sample sizes are shown below each graph. The Wilcoxon–Mann–Whitney test was used for statistical analysis. *p < .05; ***p < .001. [file GTC-28-433-s002.pdf]

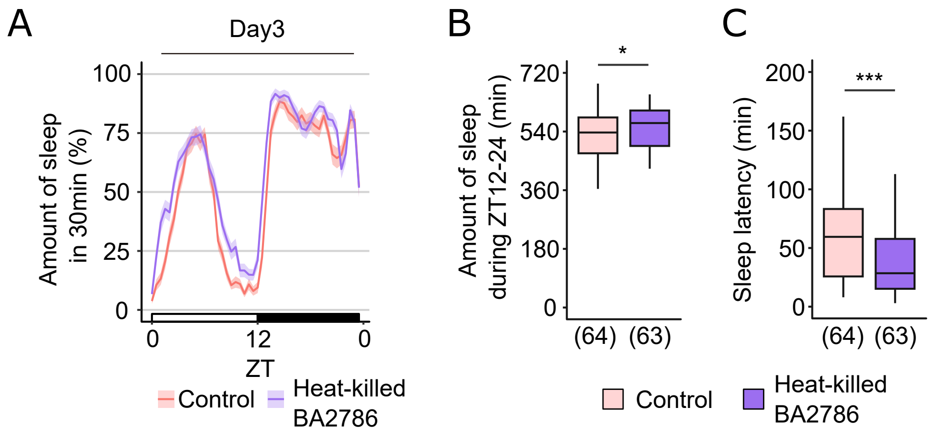

Figure S2. Heat-killed SBT2786 promoted sleep in flies. The effects of heat-killed SBT2786 were tested in flies. (A) Sleep patterns of flies on the third day. Sleep patterns are indicated as mean ± SEM. (B) Amount of sleep during ZT12–24 on day 3 and (C) sleep latency on the third day. The sample sizes are shown below each graph. The Wilcoxon–Mann–Whitney test was used for statistical analysis. *$p < 0.05$; ***$p < 0.001$.
