# Supplementary material for: Behavioral screening of sleep‐promoting effects of human intestinal and food‐associated bacteria on Drosophila melanogaster
Source: Genes Cells. 2023 Mar 28;28(6):433–46. doi: 10.1111/gtc.13025 (PMC11447928; doi:10.1111/gtc.13025)
Supplement: Supplementary file 3 — Figure S3. Food consumption by flies of BA2786 and BA003 food was not different from that of the control food. The amount of food consumed by flies in 6 h was measured for each type of food. Bars represent mean ± SD. n = 5 for each group. The Tukey–Kramer test was applied for statistical analysis. n.s.; not significant. [file GTC-28-433-s001.pdf]

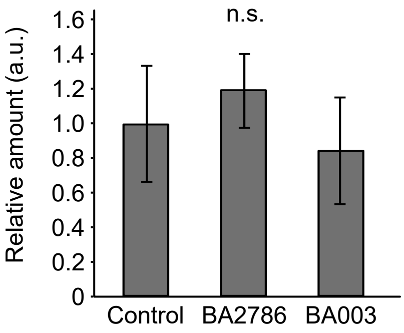

Figure S3. Food consumption by flies of BA2786 and BA003 food was not different from that of the control food. The amount of food consumed by flies in 6 h was measured for each type of food. Bars represent mean ± SD. $n = 5$ for each group. The Tukey–Kramer test was applied for statistical analysis. n.s.; not significant.
